# The Influence of Partnership Status on Fertility Intentions of Childless Women and Men Across European Countries

**DOI:** 10.1007/s10680-023-09664-5

**Published:** 2023-07-03

**Authors:** Nadia Sturm, Judith C. Koops, Roberta Rutigliano

**Affiliations:** 1 Centre for Sociological Research, KU Leuven, Leuven, Belgium; 2 Radboud University Nijmegen, Nijmegen, Netherlands; 3 Department of Sociology and Social Work, University of the Basque Country (UPV/EHU), 48940 Leioa, Spain; 4 Ikerbasque, Basque Foundation for Science, Bilbao, Spain

**Keywords:** Fertility intentions, Partnership status, Cross-national, Europe, Theory of conjunctural action

## Abstract

The absence of a suitable partner is the most frequently given reason for unmet fertility intentions across European countries while having a partner is positively associated with the intention to have a child. However, once this relationship is framed within a life-course approach, existing evidence is mixed and inconclusive. The norm to have children within a stable relationship and norms regarding the timing of childbirth are acknowledged in many contemporary societies. Therefore, the presence of a partner might have a stronger effect on fertility intentions around the social deadline for fertility, which could explain the mixed findings in previous research. This article analyses how fertility intentions are influenced by partnership status and how this relationship varies by age and across countries. We use data from the first wave of the Generations and Gender Survey to analyse a sample of childless men and women aged 18–45 years from 12 European countries. We implement logistic regression models to investigate the influence of having a partner on fertility intentions during the life course. Previous studies found that the positive influence of having a partner either decreases across the life course or does not vary significantly. This study reveals that the positive association between partnership and fertility intentions increases from the age of 18, proving that whether someone is in a partnership becomes more influential at later stages in life. After a certain age threshold, which varies across countries and gender, this positive association either turns insignificant, remains positive, or reverses.

## Introduction

Despite significant fluctuation of fertility trends and the emergence of alternative partnership arrangements at the macro-level, there is little evidence that preferences towards family formation have changed much at the micro-level. In terms of fertility, the mean ideal family size has not declined below two children per woman in Europe (Sobotka & Beaujouan, 2014) and studies on parenthood and partnerships suggest that individuals tend to follow the norm of having children within a stable relationship (Holland, 2013; Lappegård & Noack, 2015; Rutigliano & Esping-Andersen, 2018). In consequence, the absence of a partner can act as a constraint to fulfilling fertility intentions. Across European countries, a lack of the right partner for raising children is the most frequently given reason for not meeting fertility expectations (Testa, 2007). How big of a role partnership status plays when it comes to fertility intentions is, therefore, a crucial question. Since norms in terms of the timing of childbirth exist (Billari et al., 2011), having a partner might especially influence fertility intentions when nearing the social age deadlines for parenthood but have a small or no influence around the age of 18 when becoming a parent is less likely to be planned soon. Following this argumentation, an important factor likely to influence the relationship between fertility intentions and partnerships is the age of an individual.

According to prior research, being in a relationship is associated with an increase in fertility desires (Gray et al., 2013; Iacovou & Tavares, 2011; Wagner et al., 2019). Meanwhile, fertility intentions seem to decline steadily over the life course (Iacovou & Tavares, 2011; Gray et al., 2013; Liefbroer, 2009), indicating that the influence of having a partner varies with age. However, once an interaction between age and partnership status is considered, existing evidence is mixed and inconclusive ranging from no significant association over the life course (Iacovou & Tavares, 2011) to a negative association at higher ages (Liefbroer, 2009). These findings contradict the theoretical expectation that the influence of partnerships is increasing until the socially acceptable age at first childbirth. Furthermore, previous studies which accounted for an interaction between age and partnerships focussed on a single country (Iacovou & Tavares, 2011; Liefbroer, 2009), while the relation between partnerships, fertility intentions and age might vary across countries. In fact, country-level factors such as family policies and gender equality norms influence fertility levels (Esping-Andersen & Billari, 2015; Gauthier, 2007) and might soften the burden of single parenthood (see e.g. Pollmann-Schult, 2018) while loosening the positive association between the selection into a stable partnership and fertility intentions.

This study, therefore, aims at analysing the association between partnership status, age and fertility intentions across different countries. Specifically, we answer the following research question: *Are fertility intentions associated with partnership status and, if so, does this association vary by age and by country?*

Based on the theory of conjunctural action (Johnson-Hanks et al., 2011b), we hypothesise that being in a relationship is positively associated with the intention to have children and that the association increases with age as individuals near the socially acceptable ages at first childbirth. To address our research question, we apply logistic regressions to a harmonized data set from the first wave (2002–2013) of the Generations and Gender Survey (GGS). The sample consists of 22,703 childless respondents between the ages of 18–45 from 12 Northern, Western, Eastern and Southern European countries.

This study adds two main contributions to prior literature. First, we carry out a novel in-depth analysis of how the association between partnership status and fertility intentions varies during the life course. Second, we compare how this relationship varies across 12 countries while previous studies have focused only on single countries (e.g. Iacovou & Tavares, 2011; Liefbroer, 2009).

## Background

### Fertility Intentions, their Roots and the Role of Partnerships

Following Malle et al. (2001) and Bachrach and Morgan (2013, p. 460), we define intentions as “complex mental states in which there is a desire for some outcome, a belief that taking a particular action will lead to that outcome, and some degree of commitment to perform the action”. Fertility intentions can be differentiated between short-term and long-term fertility intentions (Balbo et al., 2013). In this study, we combine these two types of fertility intentions in order to analyse the overall intention to become a parent at some point during the life course. Furthermore, we focus on the fertility intentions of *childless* individuals since we are interested in studying the transition to parenthood. In contrast to subsequent births, decisions on first childbirth are to a lesser extent guided by rational considerations and institutional constraints (Harknett et al., 2014) and therefore might reflect the value attached to partnership and parenthood more clearly.

According to the theory of conjunctural action, fertility intentions are situated within a net of interdepending life domains such as working and partnering (Morgan & Bachrach, 2011). Incorporating concepts from social, psychological and biological sciences (Johnson-Hanks et al., 2011b), the theory of conjunctural action analyses the interdependence of fertility intentions, partnership and age through the lens of socially shared schemas and specific structures. Schemas are relatively stable, abstract representations of objects or events, that are automatically produced and guide day-to-day as well as future behaviour. The repeated exposure to schemas has a crucial influence on intention formation since “they become the taken-for-granted baseline assumptions for intention formation” (Bachrach & Morgan, 2013, p. 468). Partnering, becoming a parent or having a successful career are goals that are justified and motivated by a range of interconnected schemas, which characterize these events as part of the normative life course (Johnson-Hanks et al., 2011a, p. 75). Previous research shows that the norm to have children within a relationship is acknowledged by most individuals in contemporary developed societies (Holland, 2013; Lappegård & Noack, 2015). Furthermore, according to the theory of conjunctural action, being in a relationship creates a structure that is connected to family life and parenthood and might therefore activate said schemas (Bachrach & Morgan, 2013). In summary, positively valued schemas of having children within a stable relationship exist and the schema of parenthood can be activated when entering a relationship.

The majority of previous studies show a positive relationship between partnership status and intended as well as actual fertility (Berrington, 2004; Harknett & Hartnett, 2014; Kapitány et al., 2012; Rybińska & Morgan, 2019). Single men and women are significantly more likely to postpone childbirth and abandon child-bearing plans in comparison to married and cohabiting respondents in the Netherlands, Switzerland, Hungary and Bulgaria (Kapitány et al., 2012). Berrington (2004) finds that having a partner increases the odds of actually giving birth for women in Great Britain (Berrington, 2004). On a country-level, across 22 European countries, the proportion of women who realize their birth intentions is positively associated with the proportion of childless *partnered* women who plan a birth but not with the proportion of childless, *single* women planning childbirth (Harknett & Hartnett, 2014). Furthermore, people who never had a partner or were in several living-apart-together relationships during their life course are most likely to remain childless in Germany (Raab & Struffolino, 2020) and Finland (Saarela & Skirbekk, 2020). In conclusion, the clear positive association between partnerships and fertility intentions continues to persist in recent years and across cultural settings.

### Partnership and Fertility Intentions Over the Life-Course

Having a partner is likely to influence fertility intentions positively (Harknett & Hartnett, 2014; Kapitány et al., 2012; Berrington, 2004; Rybińska & Morgan, 2019), but it is still unclear whether the strength of this influence varies during the life course. According to the theory of conjunctural action, the influence of the current structure on intentions is weaker if the intention refers to behaviour that is situated in the far future (Bachrach & Morgan, 2013). Since norms regarding the suitable age at childbirth exist (Billari et al., 2011; Liefbroer & Billari, 2010), it seems likely that the influence of having a partner on fertility intentions increases during the life course. Fertility intentions of singles and people in a relationship might not differ as strongly in their early twenties when becoming a parent lies further in the future, compared to in their thirties, when the intention to become a parent is closer to potentially being realized.

In terms of the main effect of age on fertility outcomes, studies find that, on average, fertility desires and the expected number of children decline steadily during the life course (Gray et al., 2013; Liefbroer, 2009; Iacovou & Tavares, 2011). However, once an interaction between age and partnership status is considered, existing evidence is mixed and inconclusive. For Great Britain, Iacovou and Tavares (2011) do not find significant interaction effects between partnership status and age on fertility intentions and conclude that partnership variables do not have a greater effect on changes in expectations towards the end of the fertile years. In contrast, for the Netherlands, Liefbroer (2009) finds that the difference in the expected number of children between partnered and single respondents increases with age: as people grow older, the expected family size declines across both groups, but the decline is most pronounced for people without a partner. This finding supports the assumption that partnership status has a larger effect as people grow older. However, results change when controlling for the number of children. Over time, the difference in mean expected family size of *childless* married and unmarried respondents becomes smaller (Liefbroer, 2009), meaning that the influence of having a partner actually decreases. A possible explanation could be the increasing selectivity of the group of childless people. If someone remains childless until a certain age, they might never have considered or no longer consider having children—independently from having a partner or not.

### Partnership and Fertility Intentions: Differences on Country-Level

Due to different institutional and cultural settings, the overall influence of partnerships on fertility intentions as well as changes in this relationship during the life course might vary across countries. First, family policies and welfare systems could influence the relationship between partnerships and fertility intentions by supporting the fertility desire of single would-be parents. Previous studies, for example, suggest that higher financial benefits and childcare provision increase the life satisfaction of single mothers (Pollmann-Schult, 2018) and reduce lone mothers’ poverty risk (Misra et al., 2012; Hübgen, 2018). Secondly, norms and attitudes towards having and raising a child within a relationship or outside differ across countries and might mediate/moderate the role of a partner in fertility decisions. According to Liefbroer et al. (2015), approval of fertility behaviour—such as the relationship status at birth of a child—varies strongly across countries and influences individual-level attitudes. Thus, in countries with more liberal attitudes towards childbirth outside of a union, partnership status might have a lower influence on fertility intentions.

In general, previous studies show a positive effect of having a partner on fertility intentions in various countries (Harknett & Hartnett, 2014; Kapitány et al., 2012), indicating that the influence is significant across different social contexts. However, due to the low prevalence of births outside of a union (Sobotka & Toulemon, 2008), research analysing the role of society or family policy on the relation between fertility intentions and having a partner *at all* is limited. A recent study by Jirjahn and Chadi (2020) compares the likelihood of childbearing while being single in East and West Germany. According to their results, women in East Germany are more likely to realize a planned pregnancy without a partner than women in West Germany. As this finding persists after controlling for non-marital fertility, economic factors and availability of childcare, the authors conclude that more egalitarian gender role models in East Germany are the main explanation for the East–West difference (Jirjahn & Chadi, 2020). These findings indicate that the association between partnerships and fertility intentions might vary according to the prevalence of gender egalitarian attitudes in different cultural settings.

Possibly, the association between fertility intentions and partnerships, therefore, follows similar patterns across different welfare and value systems. One way to classify countries, which has been applied to fertility research (see e.g. Rutigliano, 2020; Del Boca et al., 2009) is, for example, offered by Gauthier (1996), who groups countries along two dimensions: family policies and social values. Gauthier’s classification system is particularly suitable since it focuses specifically on the provision of childcare rather than other forms of care. She identifies four different regimes across Western Europe. For the countries in our sample, the following three apply: pro-traditional (in our sample, Austria, Germany and Italy), pro-egalitarian (in our sample, the Netherlands, Norway and Sweden) and pro-family/pro-natalist (in our sample, Belgium and France).

Given the previous theoretical arguments and empirical evidence, we expect that the relationship between partnership and fertility intentions is positive and significant across all countries, although the strength of the relationship might vary. We would expect that the influence of having a partner on fertility intentions is bigger in countries with a pro-traditional regime (Austria, Germany, Italy), where a traditional male-breadwinner model persists, as well as in the Eastern European countries in our sample (Bulgaria, Hungary, Lithuania, Romania), where participation of women in the labour force was strongly encouraged during communism (Pollert, 2003), but where the declining provision of childcare and higher investment in parental leave indicate a form of re-traditionalization (Pascall & Manning, 2000). This would be followed by the pro-family/pro-natalist regime in Belgium and France and, finally, the pro-egalitarian regimes in the Netherlands, Norway and Sweden, where dependence on a partner might be lowest due to more universal provision of childcare and higher gender equality. Furthermore, we expect that the influence of partnerships *during the life course* also varies considerably across countries. Since norms regarding the ideal age at first birth vary across countries (Liefbroer et al., 2015), the association between fertility intentions and partnerships might be stronger or weaker at different ages across countries.

### Further Influences on Fertility Intentions: Gender and Educational Level

Since norms and attitudes towards childbearing, childlessness and partnerships affect both women and men (Hadley, 2017), a positive influence of having a partner on fertility intentions across gender can be expected. However, the relation between age and fertility intentions could vary by gender, since women’s fecundity declines earlier with age (Iacovou & Tavares, 2011) and socially acceptable age limits for childbearing are lower for women than for men (Liefbroer et al., 2015). Still, if men reach a certain age, the majority of women with which they could have children would also be in a similar age group (Iacovou & Tavares, 2011).

Socio-economic background is considered to have an influence on the partnership status at first birth (Koops et al., 2017; Perelli-Harris et al., 2010). A higher socio-economic background increases the likelihood of having a child within a stable partnership and decreases the likelihood to become a single mother (Koops et al., 2021). Analysing a possible variation due to socio-economic status goes beyond the scope of this paper, but is controlled for in the analysis.

## Hypotheses

The current study aims to investigate how fertility intentions are influenced by partnership status and whether this relation varies by age and across countries. Based on the theory of conjunctural action and previous empirical findings we first hypothesise that *childless men and women in a relationship are more likely to intend to have a child or children than people who are not in a relationship (H1)*. The second hypothesis follows the theory of conjunctural action and assumes that being in a relationship (the current structure) has a stronger influence on becoming a parent (intention formation) at ages closer to the normative age at first childbirth. Compared to the start of the observation period (at 18), *the association between partnership status and fertility intentions should be stronger with increasing age (H2)*. Based on previous cross-country studies we further expect the influence of having a partner on fertility intentions to be significantly positive across countries, despite a possible variation in both the effect size and across the life course.

## Data and Method

### Data and Sample Selection

The analyses are based on a data set from the first wave of the Generations and Gender Survey (consolidated GGS data set, version 4.3.1) which contains harmonized cross-sectional data on fertility intentions and behaviour as well as partnership status (Gauthier et al., 2018; Vikat et al., 2007). For the sake of comparability, our sample consists of all childless men and women between the ages of 18 to 45, who are not pregnant at the time of the interview and who do not report any fecundity problems. Our final country selection consists of 9 countries in which both women and men were included, three countries in which the data for men had to be excluded (Norway, Belgium and Germany) and one country in which the data for women was excluded (Russia). To obtain reliable results, we excluded countries with too many missings on the dependent variable and countries in which, for example, only partnered respondents were asked about their fertility intentions since this would bias our results. We, therefore, excluded data for men in Poland, Norway, the Czech Republic, Belgium and Germany. Among the women, data from Russia, the Czech Republic and Poland were excluded due to high missings and biased data.^1^ Due to too low sample sizes in Georgia and Estonia, the data for both men and women were excluded.

Table 1 provides an overview of the sample size across countries and gender as well as the year of data collection. Due to data limitations, same-sex partnerships had to be excluded from the analysis. After this sample selection, we end up with a final sample of 22,703 respondents.

**Table 1:** Information on the datasets: year of collection and sample size. *Source* Generations and Gender survey, wave 1. The sample includes childless men and women at age 18–45

Country	Year	Sample size ^a^		
		Total	Women	Men
Scandinavian countries				
Norway	2007–08	1,203	1,203	–
Sweden	2012–13	2054	937	1117
Western European countries				
Belgium	2008–10	614	614	–
Austria	2008–09	2119	1105	1014
Germany	2005	657	657	–
France	2005	1936	1037	899
Netherlands	2002–04	1481	758	723
Eastern European countries				
Bulgaria	2004	2814	1246	1568
Hungary	2004–05	2,585	1061	1524
Romania	2005	1762	604	1158
Lithuania	2006	1,967	796	1171
Russia	2004	792	–	792
Southern European country				
Italy	2003–04	2746	1248	1498

^a^ Sample size after deleting respondents with values missing on the (in)dependent variable(s)

### Dependent Variable

To measure overall fertility intentions, we combine short-term and long-term intentions. This makes the fertility intentions of younger and older respondents comparable. Individuals in the GGS are asked (1) whether they intend to have children within three years, to which they can answer on a scale from 1 to 4 (definitely not, probably not, probably yes, definitely yes). In case they probably or definitely do not intend to have a(nother) child, they are asked (2) whether they intend to have children at all, to which they can answer by choosing between the same answer categories. For the sake of clarity and due to diverging answer categories in three countries^2^, we dichotomize the four answer categories. We re-categorize the questions in the following way: If respondents intend to have children *either within or after three years* they are considered as intending to have children (positive fertility intention). If they do not intend to have children *neither before nor after three years,* they do not intend to have children (negative fertility intentions).

In some countries, the question of fertility intentions also explicitly covers the intention to adopt. Therefore, both the intention to have a child and the intention to adopt a child are included in the final variable to give a more accurate reflection of the data. Nevertheless, only 1.3% of the total sample of individuals mention that they probably or definitely intend to adopt a child.

### Other Variables

The variable *partnership status* measures whether respondents are in a relationship (married, non-marital cohabitation, living-apart-together) or not at the time of the interview. Due to the relatively low number of childless people and the resulting low sample size within the sub-groups, it is not possible to distinguish between all different types of relationships. Instead, partnership status is a dichotomous variable distinguishing between people in a relationship (married, cohabiting, LAT) and singles. For the descriptive statistics, *age* of respondents is recoded as a categorical variable (“below 25”, “25–29”, “30–34”, “above 34”). In this way, we address the nonlinearity of fertility intentions across different stages of the life course. For the models, age is measured as a continuous as well as squared variable. Due to the small sample size, this strategy allows for exploring the interaction between partnership status, fertility intentions and age. We control for socio-economic background, as women with a higher socio-economic background are more likely to have a child within a stable partnership (Koops et al., 2021). In our study socio-economic background is measured by a continuous scale capturing the *highest educational level* (Brons & Mooyaart, 2018) based on the ISLED Score (Schröder & Ganzeboom, 2014).

### Analytical Strategy

To test our hypotheses, we implement logistic regressions in which the dependent variable is one if an individual intends to have a child and zero otherwise. The main independent variables are whether or not someone has a partner at the time of the interview and the age of the respondent. We further control for highest educational level (measured continuously).

We present *average marginal effects* (AMEs), *predicted probabilities* and *differences in probability* separately by country and by gender. When calculating the predicted probabilities and difference in probability we include an interaction effect between partnership status and age in the model to explore the variation of the effect of partnership status in more detail.

## Results

This section illustrates the results of the descriptive (5.1) and the multivariate analysis (5.2). Due to limited space and data limitations (i.e., we cannot compare results for women and men across all countries due to missing data), we present the representative results for women only. Completed results for men are available in Tables 4, 5, 6, 7, 8, 9, 10 in the Appendix. In the last paragraph (5.3), we compare the findings for men and women where applicable.

### Results from the Descriptive Analysis

Table 2 contains the share of childless women intending to have a child across countries, age groups and partnership status. Since the number of respondents across age categories is not distributed equally, the results have to be interpreted with caution. Nevertheless, they give a first impression of the association between age, partnership status and fertility intentions.

**Table 2:** *Share of respondents intending to have children* across countries, age groups, and partnership status and *share of ever-partnered among respondents*: Women. *Source* Generations and Gender survey, wave 1; own calculations

Age	Country	No partner		Partner		Ever cohab	Country	No partner		Partner		Ever cohab
		%	N	%	N	%		%	N	%	N	%
Scandinavian countries												
< 25	NOR	89.3	269	93.6	277	6.2	SWE	90.6	241	93.1	244	7.6
25–29		81.5	93	93.5	174	13.7		87.3	62	94.4	136	14.9
30–34		82.8	53	89.4	76	17.5		79.3	23	91.6	66	13.9
> 34		27.4	20	41.6	35	22.3		50.0	15	34.9	22	18.3
Ø		78.8	435	86.3	562	11.6		86.1	341	86.5	468	11.0
Western European countries												
< 25	AUT	88.4	176	92.1	235	4.0	BEL	82.0	100	84.2	144	2.7
25–29		87.7	79	90.1	193	6.6		68.9	31	90.2	83	9.5
30–34		81.5	31	83.6	87	17.0		63.6	14	75.5	37	8.5
> 34		43.5	34	43.3	55	21.5		20.8	10	24.6	16	20.4
Ø		79.0	320	81.4	570	9.6		65.4	155	74.3	280	8.1
< 25	FRA	74.0	165	83.4	246	6.0	DEU	81.3	100	92.6	137	4.1
25–29		79.5	58	94.2	131	11.8		90.0	45	85.1	80	5.6
30–34		91.1	51	85.0	51	25.0		55.6	20	79.6	43	5.6
> 34		32.0	33	39.8	35	24.6		17.0	10	15.1	14	9.9
Ø		67.5	307	79.6	463	12.7		65.3	175	70.4	274	5.9
< 25	NLD	89.5	96	95.8	114	4.7						
25–29		88.0	59	93.9	140	9.7						
30–34		78.5	44	74.0	74	15.4						
> 34		19.2	15	17.2	16	21.1						
Ø		68.7	204	74.6	344	12.0						
Eastern European countries												
< 25	HUN	93.4	228	96.3	183	2.8	BGR	93.4	454	99.1	214	0.6
25–29		92.1	140	98.0	239	5.1		91.5	129	99.2	124	2.3
30–34		88.1	59	92.3	72	17.2		89.0	65	90.0	45	2.4
> 34		40.0	20	55.6	20	18.6		54.7	52	76.7	46	5.2
Ø		87.1	447	93.8	514	6.9		88.1	700	95.1	429	1.7
< 25	LTU	98.8	336	99.3	157	1.6		96.3	181	98.7	77	0.4
25–29		96.8	61	100	69	6.8		92.9	52	98.7	74	0.8
30 –34		91.2	31	95.8	23	12.1		87.9	29	95.4	62	8.2
> 34		26.3	20	31.2	10	7.4		46.4	26	50.9	27	14.7
Ø		87.3	448	91.5	259	4.0		86.5	288	88.6	240	4.3
Southern European country												
< 25	ITA	88.3	205	98.0	150	0.3						
25–29		93.9	108	97.9	144	2.3						
30–34		84.7	78	95.6	110	4.4						
> 34		37.2	81	53.9	95	11.2						
Ø		71.8	472	84.4	499	4.8						

Across all countries, the share of childless women intending to have children (averaged across age groups) is higher among those in a partnership, which points to a positive association between being in a partnership and intending to have children. How large the difference between singles and partnered women is, varies across countries: from about 10 percentage points in Italy, France and Belgium to only about 0.5 percentage points difference in Sweden and Austria. Interestingly, the descriptive results show that the overall share of people intending to have children is higher in Italy and Northern and Eastern European countries than in Western European countries.

In terms of fertility intentions across age groups, the descriptive results clearly show that the share of people intending to have children is lower in the age group 34–45. In many, but not all countries, the percentage intending to have children is highest among the respondents below 25. Especially in the age group over 34 years, the difference between people in a partnership and singles is more pronounced in many countries. Outliers exist though: in Austria, Germany and the Netherlands, the shares among this age group do not differ much. In Sweden, the share of people intending childbirth is even higher among singles in the oldest age group.

### Results from the Multivariate Analysis

#### Partnership Status and Fertility intentions: Average Marginal Effects

From the logistic models, we are calculating the AMEs of having a partner on the intention to have a child. The AMEs represent the averaged difference in probability of fertility intentions if someone has a partner, given that the values of the covariates age^3^ and education remain at their observed levels. For example, for partnered women in Norway, the likelihood of intending to have a child increases on average by 7.5 percentage points compared to single women.

**Table 3:** Average marginal effect of having a partner on fertility intentions (with age as squared and continuous variable): Women. *Source* Generations and Gender survey, wave 1; own calculations

Coun.	Partner	95%-Confidence interval	
Scandinavian countries			
NOR	0.075***	0.0382	0.111
SWE	0.031	− 0.010	0.072
Western European countries			
AUT	0.021	− 0.022	0.064
BEL	0.068*	0.038	0.131
DEU	0.067*	0.011	0.124
FRA	0.072**	0.024	0.120
NLD	0.022	− 0.025	0.070
Eastern European countries			
BUL	0.068***	0.041	0.095
HUN	0.044**	0.013	0.075
ROU	0.057**	0.016	0.099
LIT	0.029*	0.002	0.056
Southern European countries			
ITA	0.113***	0.078	0.148

Average marginal effects

+ *p* < 0.10, *p < 0.05, **p 0.01, *** *p* < 0.001

Across all countries, except for women in Austria, the Netherlands and Sweden, having a partner is positively associated with the intention to have children. For women in Norway, the likelihood increases by 7.5 percentage points. Among the Western European countries, the largest differences across partnership status are found in France (7.2 percentage points) and Belgium (6.8 percentage points). Across Eastern European countries, the increase varies between 2.9 percentage points in Lithuania to 6.8 percentage points in Bulgaria. Across all countries, the highest increase in the likelihood of intending to have children can be observed for women in Italy (11.3 percentage points) and Norway (7.5 percentage points) and the lowest in Lithuania (2.9 percentage points), followed by Hungary (4.4 percentage points). Surprisingly, the influence of having a partner does not yield significant results in Sweden, Austria and the Netherlands. We do not find a specific trend in terms of the four different family policy regimes.

In general, the results confirm that having a partner influences the intention to have children positively (H1). However, within the geographical regions as well as when we consider different welfare regimes, the coefficients vary considerably (Table 3).

#### Partnerships and Fertility Intentions During the Life Course: Predicted Probability and Difference in Probability

In this section, we present the predicted probability and the difference in probability of fertility intentions for childless women with and without a partner across different ages.^4^ The predicted probability does not tell us whether the differences we observe between partnered and single women in terms of their fertility intentions are statistically different. We therefore also calculate the difference in probability to analyse whether the groups differ significantly from each other.

**Fig. 1:**
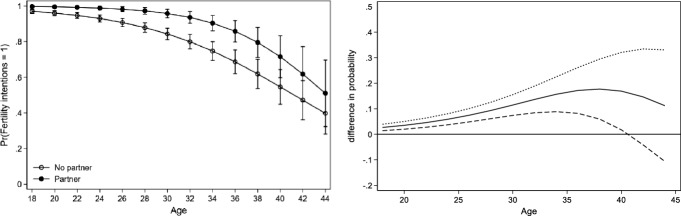
Predicted probability of intending to have children (left) and difference in probability of fertility intention when being in a relationship (right): Women in Bulgaria. *Source*: Generations and Gender survey, wave 1; own calculations

**Fig. 2:**
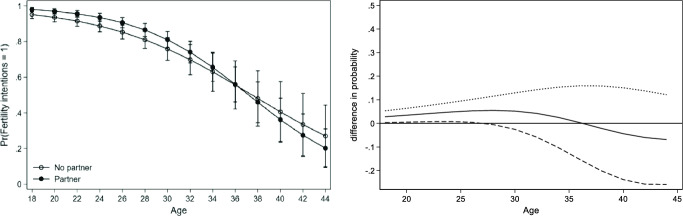
Predicted probability of intending to have children (left) and difference in probability of fertility intention when being in a relationship (right): Women in Sweden. *Source*: Generations and Gender survey, wave 1; own calculations

**Fig. 3:**
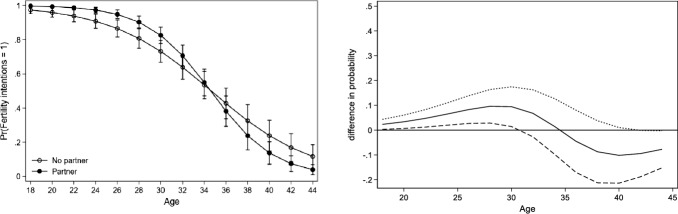
Predicted probability of intending to have children (left) and difference in probability of fertility intention when being in a relationship (right): Women in the Netherlands. *Source*: Generations and Gender survey, wave 1; own calculations

After running separate models for each country, we observe two main patterns of the association between fertility intentions, partnership status and age. For the sake of brevity, we present the results of Bulgaria, Sweden and the Netherlands as representatives of the two observed patterns. A complete overview of the predicted probability and difference in probabilities across all countries and both genders can be found in the appendix (see Table 10 in Appendix). For each figure of the three countries (Figs. 1, 2, 3), we present two graphs. The graph in Panel A displays the conditional predicted probability of fertility intentions for partnered (in black dots) versus single childless women (white dots). The graph in Panel B displays the difference in probability between the two groups and their 95% confidence intervals (dotted curve), with partnered women represented by the solid curve and single women as a reference group. A curve and its confidence intervals above zero indicate that partnered women have a significantly higher probability of intending to have children than single women. Once the lower confidence interval moves below zero, single and partnered women do not differ significantly from each other. The predicted probability is displayed at the mean level of education within each country.

Before turning to the more specific results across the two patterns, several general results on the non-linear association between fertility intentions, partnership status and age should be discussed. Firstly, the overall probability to intend to have children decreases during the life course for both partnered and single women (see Panel A in Figs. 1, 2 and 3). Secondly, with the exception of Austria, childless women with a partner have a higher probability of reporting positive fertility intentions than those without a partner (see for example Fig. 1, Panel B). This finding holds even if the difference between partnered and single people is very small (Fig. 2, Panel B) or if the relation reverses at some point (Fig. 3, Panel B). Thirdly, across all countries, the difference between partnered and unpartnered women in terms of fertility intentions turns insignificant at some point. Countries vary though in terms of the strength of the positive association between partnership status and fertility intentions as well as in terms of the age point at which the difference between single and partnered women turns insignificant or reverses. This leads us to a closer observation of the two previously mentioned patterns.

The *first pattern* is characterized by a pronounced difference between partnered and single women in terms of their fertility intentions which does not reverse at older ages. This pattern is represented by Bulgaria in Fig. 1. The *second pattern* is characterized by a smaller positive influence of having a partner on fertility intentions which, in most cases, turns insignificant between the age of 25–35, as represented by Sweden (Fig. 2). At older ages, countries in this pattern show signs of a reversal of the association. This means that after a certain age, single women are even more likely to intend to have children than partnered women. For the Netherlands (see Fig. 3) the reversal of the association reaches statistical significance. Below, we analyse the two patterns and the countries in which they appear in more detail.

##### Pattern 1

A pronounced influence of having a partner on fertility intentions which tends to strengthen between the ages 30 to 35, is primarily found for women in Bulgaria, Romania, Norway and Italy. The influence of having a partner remains significant and positive until older ages. The difference between partnered and single people is no longer significant at the age of 40 in the case of Bulgaria (Fig. 1, Panel B) and slightly before the age of 40 in Norway and Romania. Finally, in Italy, the difference is no longer significant between the ages 40–45. A less pronounced but also positive association, which does not reverse, can be found in Belgium, France and Lithuania. For women in all countries, except for Austria, a significant positive association between having a partner and fertility intentions is found at some point in the life course.

##### Pattern 2

A smaller positive association between partnership and fertility intentions which shows signs of a reversal, is found for women in Sweden, the Netherlands, Germany and Hungary (for Austria, signs of a reversal but no significantly positive association can be found). According to the predicted probability (Fig. 2, Panel A), Swedish women with a partner have a higher probability of intending to have children until about the age of 36. The difference in probability (Fig. 2, Panel B) shows that the fertility intentions of singles and partnered women only differ slightly and turn insignificant already between the age of 25–30. Having a partner does have a slightly positive influence on Swedish women but the difference to single women is very small and can only be observed before the age of thirty.

For the above-mentioned countries, a reversal in the association between partnerships and fertility intentions is found at older ages. The predicted probability of intending to have a child among singles surpasses that of people in a relationship approximately at the age of 35 or above—but this finding is only significant for Dutch women after the age of 42–44 (Fig. 3). While the influence of having a partner among Dutch respondents does increase earlier in the life course, it decreases and even reverses later, meaning that single respondents are more likely to intend to have children than partnered people after the age of 42.

### Additional Findings on Fertility Intentions Across Gender

A comparison of representative findings for men and women can be made in 9 out of the 12 countries in our data set. In the following, we shortly discuss our findings in terms of a difference in the relation between partnerships and fertility intentions across gender.

On the one hand, the overall average influence of having a partner on the probability of intending to have children yields comparable results for men and women. For example, the effect is insignificant for both genders in the Netherlands and significantly positive among both genders in the remaining countries. On the other hand, the predicted probability and differences in probability show that there are considerable differences in the effect of partnership on fertility intentions across gender when a variation by age is taken into account. In Sweden, Hungary and Lithuania, the differences between singles and people in a relationship turn insignificant later for men than for women. In France, the Netherlands, Romania and Italy the association between partnership and fertility intentions turns insignificant—or in the case of the Netherlands reverses—at relatively similar age points for childless men and women. Men in Bulgaria are the only group across the sample in which the effect of having a partner remains significantly positive across the entire observed age period. In Austria, the effect of having a partner is not significant at any point for women, while the difference is significant for men until approximately the ages of 30–35.

### Summary of Results

The relationship between partnership status and fertility intentions varies strongly with age. For women across all countries, except for Austria, the positive association between partnership and fertility intentions increases up to a certain age, after which single and partnered women no longer differ significantly in terms of their fertility intentions or the association between single and partnered people reverses as in the case of the Netherlands. Importantly, the association turns insignificant at different age points across countries: Among women in the Western European countries and Sweden, the difference turns insignificant approximately between the ages of 30–35. An exception is Austria, where the association is not significant at any point. In Bulgaria, Romania, Italy and Norway the positive influence of having a partner turns insignificant around the age of 40 or above. In Lithuania, on the other hand, the association is only significantly positive approximately between the age of 28 to 35. In Hungary, the association also turns insignificant slightly before the age of 35, similar to the pattern in the Western European countries. Furthermore, while the descriptive analysis and the AMEs suggest a similar pattern across genders within the countries, the predicted probability and difference in probability show that the relationship between partnership status, age and fertility intentions plays out differently for men and women.

## Conclusion and Discussion

Our study analysed how fertility intentions of childless men and women are influenced by partnership status and how this relationship varies by age and across countries. To test our hypotheses, we implemented logistic regressions on data from the Generations and Gender Survey including childless men from 9 countries and childless women from 11 countries in Europe.

Based on the theory of conjunctural action, we hypothesise that *childless men and women in a relationship are more likely to intend to have a child or children than people who are not in a relationship.* We found that having a partner is positively associated with the intention to have children across all countries except for women in Austria and Sweden and men and women in the Netherlands. This result becomes even clearer when considering the varying influence of partnerships on fertility intentions at different ages: we show that partnerships—in a certain age period—are then also significantly positively related to the intention to have children for men and women in the Netherlands as well as women in Sweden. Therefore, this study confirms our first hypothesis. The results are in line with previous studies which find a positive association between partnerships and fertility intentions (see e.g. Kapitány et al., 2012; Rybińska & Morgan, 2019). By expanding this analysis to a large number of countries, we show that this finding is generalizable to a wide variety of cultural settings. From the data, we do not find support for our expectation that having a partner is particularly strongly associated with the intention to have children in the pro-traditional and Eastern European countries in our sample, rather than in the pro-family/pro-natalist and pro-egalitarian countries.

Furthermore, our results support our second hypothesis that *the influence of partnership status on fertility intentions is larger with increasing age*. Previous studies, which included an interaction between fertility intentions, partnerships and age, found that the positive influence of having a partner either decreases across the life course (Liefbroer, 2009) or does not vary significantly (Iacovou & Tavares, 2011). Our results instead reveal that partnerships become increasingly important during the life course but up to a certain age, after which the association turns insignificant. In some but not all countries, the decline in fertility intentions is less steep for men, which fits the explanation that men’s fecundity declines later and that parenthood is on average accepted until later ages.

Finally, when assessing the differences and similarities between countries, we identified two main patterns of interaction between partnership, age and fertility intentions. In the first group of countries, the difference in fertility intentions between singles and partnered people turns insignificant later, i.e., around the age of 40 or later and does not reverse. Countries in the first group are Bulgaria, Romania, Italy and Norway, also the results for men in Hungary and Lithuania correspond with this pattern. Furthermore, while the association is less pronounced for women in Belgium and Lithuania as well as men and women in France, no sign of a reversal is found for these countries. In the second group of countries, the difference in fertility intentions between singles and partnered people partly turns insignificant at an early stage of life, i.e., between the ages of 25 and 35 or shows signs of a reversal. This was true for the Netherlands, Germany, Austria and Sweden, but also for women in Hungary and men in Russia. This reversal of the association was only significant for both women and men in the Dutch sample. There, the influence of having a partner is positive, but the difference between singles and partnered people turns insignificant and then reverses after approximately the age of 40. After that age, singles even report a significantly *higher* probability of intending to have children than people in a relationship.

These findings pose two important questions: First, why does the positive association between partnership and fertility intentions, which increases from the age of 18, turn insignificant or reverses at some point? Second, why does this happen at different time points in different countries?

Concerning the diminishing difference between people with and without a partner in terms of their fertility intentions, we would assume that the presence of a partner plays an increasingly small role after a certain age. Given the so-called social age deadlines (finding yourself too old to have children) and biological deadlines (not being able to have children), the decision not to have children is more likely to be irreversible after a certain age. In fact, according to Rybińska and Morgan (2019) half of the permanently childless women repeatedly postponed childbearing before adopting childlessness expectations between the age of 35–40. Fertility intentions are then no longer significantly influenced by partnership status, although it might have been the deciding factor at younger women’s age.

The nonsignificant difference between single and partnered people could also be explained by the composition and shared characteristics of the group of older childless respondents. At later stages in life, childless people could be even more determined to fulfil their childbearing intentions, even—or especially—if they are currently not in a relationship. Findings by Wagner et al. (2019) indicate that women and men between the age of 35–37 are more polarized in terms of their fertility intentions. While the majority of women change their preferences from intending to not intending children, others are increasingly likely to report short-term fertility intentions (Wagner et al., 2019). If single, childless individuals would still like to realize their fertility intentions as they near their social and biological age deadlines, they might try to realize these intentions, e.g. by intensifying the search for a suitable partner. Furthermore, although this might apply to a relatively small group, single individuals could consider realizing their fertility intentions without a partner, e.g. by considering assisted reproductive techniques or adoption. After a certain age, older singles might then, on average, even report higher fertility intentions than older partnered people—as can be observed for the Dutch respondents—or the differences between the two groups might decrease and turn insignificant as the results for the other patterns show. Another explanation for this finding could be that the sample of older, childless respondents is highly selective and consists to a larger extent of individuals that repeatedly chose childlessness during their life course. Among *partnered,* older individuals, a larger majority might have voluntarily decided for permanent childlessness. In the group of *single,* older respondents, on the other hand, the missing partner could be the crucial constraint to the realization of their fertility intentions. Nevertheless, only a small percentage of the population is generally considered to be voluntarily childless (see e.g.Verweij et al., 2021).

Across countries, our results show that the difference between singles and partnered people in terms of their fertility intentions turns insignificant at different age points. Which factors can explain these differences? According to Merz and Liefbroer (2012), attitudes towards voluntary childlessness are more negative in Eastern and Southern European countries than in Western European countries, which was also reflected in the overall share of people intending to have children across countries (see descriptive results). In a context of lower voluntarily childlessness, firstly, respondents with a partner might, in tendency, report the intention to have children even at older ages. Secondly, being single might be the main barrier to realizing fertility intentions. For example, among women, 70% of the older, childless respondents in Lithuania and around 60% of the older, childless respondents in Bulgaria and Hungary are single while only around 35% of the respondents are single and childless at older ages in Germany, Austria and Sweden (see Appendix 4). Still, in other countries (for example for women in Romania, the Netherlands, France or Norway) the share of single and partnered people in the older age group is relatively balanced (Appendix 4). Moreover, family policies and regimes, level of voluntary childlessness or mean age at first birth do not explain differences and similarities in trends across countries consistently. The association between partnerships and fertility intentions most likely reflects a complex interplay between the above-mentioned and other factors, which might not be apparent when ordering the countries according to a single indicator. Our findings in terms of a variation of the association between partnerships and fertility during the life course have to be considered carefully since the predicted probabilities are not directly comparable across countries and the question of causality remains open. Furthermore, while it seems that the difference between partnered and single respondents turns insignificant earlier in Western than in Eastern European countries (and Italy as a Southern European country), outliers exist. More countries, data on both genders for every country and higher sample sizes of childless individuals would be needed to analyse these findings conclusively.

Some limitations have to be acknowledged. Firstly, the group of childless singles might be more heterogenous than reflected in our analysis, especially at older ages. For example, singles who cohabited at some point in their life might have more positive fertility intentions and expect to have another partner than singles who never cohabited with a partner before. Unfortunately, the sample size of childless singles is too low to accurately account for this heterogeneity. While further variables may influence the relationship between partnership and fertility intentions, the sample size and data availability limit our analysis to the variables age and education. Future research could analyse the discussed relations at different educational levels since previous studies suggest a considerable variation in the association due to socio-economic background (Koops et al., 2021). A further fruitful approach would be to include a more detailed measure of partnership status, which was not possible in our analysis because of the sample size. Due to the low prevalence of people who decide to have a child independently of a partner, an additional analysis could distinguish between people who are in a relationship and view it as a stable commitment and people who question the stability of their relationship.

Our study has shown that the influence of partnerships on fertility intentions varies greatly during the life course and that partnerships continue to be an important factor when considering parenthood across different cultural settings—even if alternative partnership arrangements emerged and having a child outside of a union is more accepted. Since the intention to become a parent and to have a child within a relationship has remained stable over time and especially in the light of postponed parenthood and decreasing fertility levels, analysing the relationship between partnership status, age and fertility intentions remains an important task for future research.

## Figures and Tables

**Table 10:**
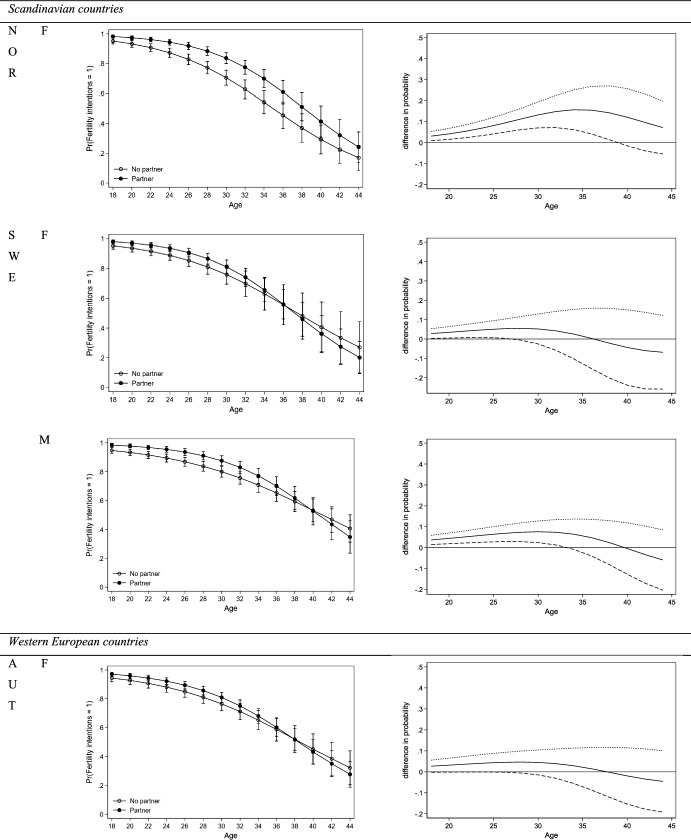

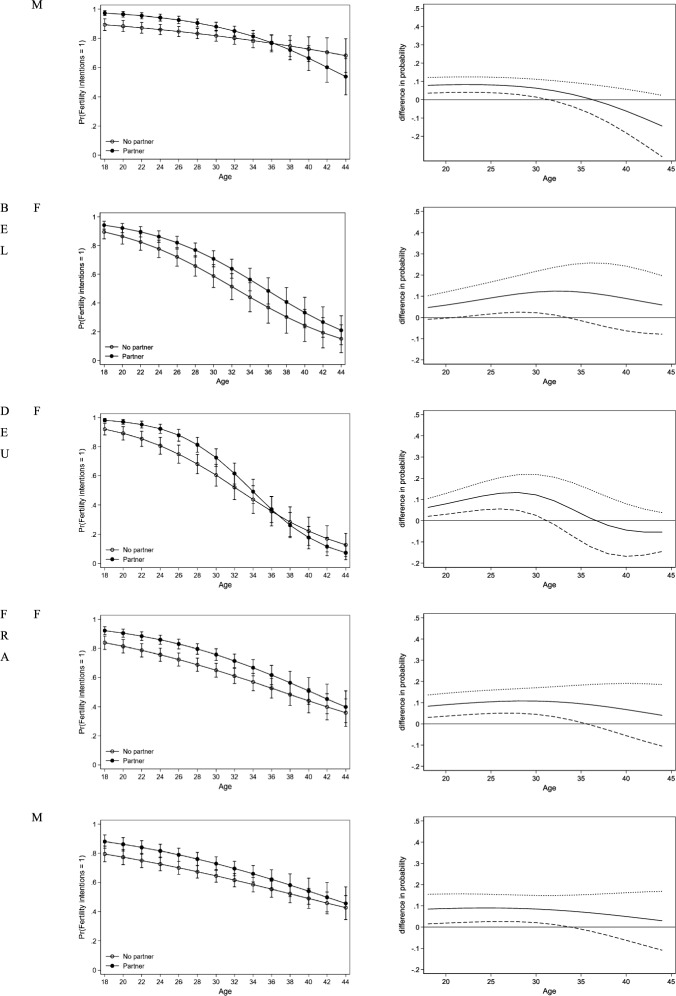

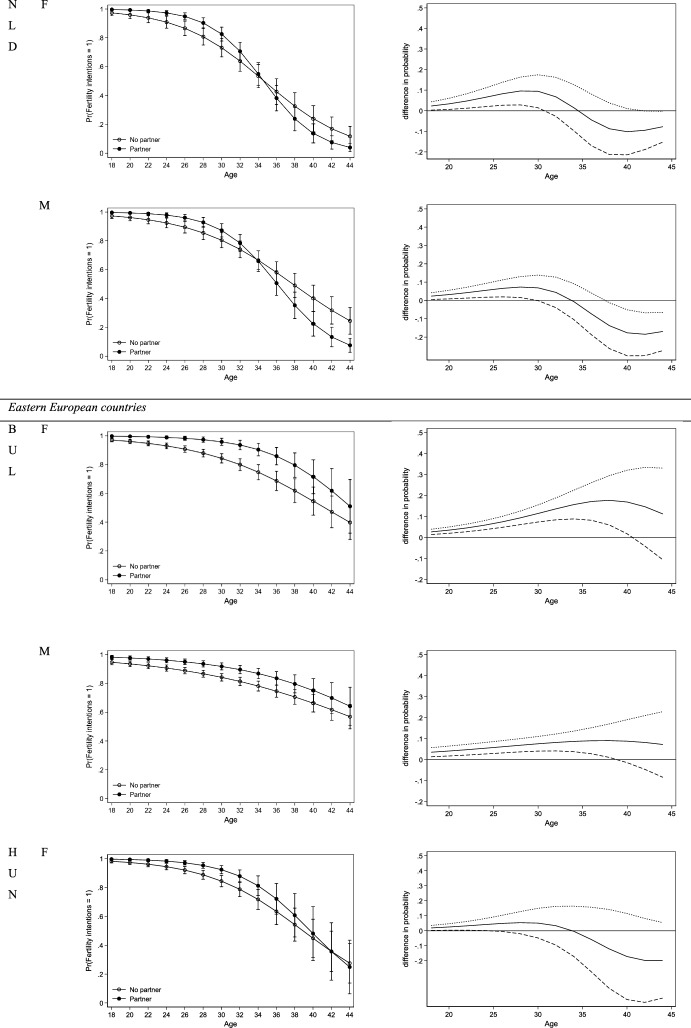

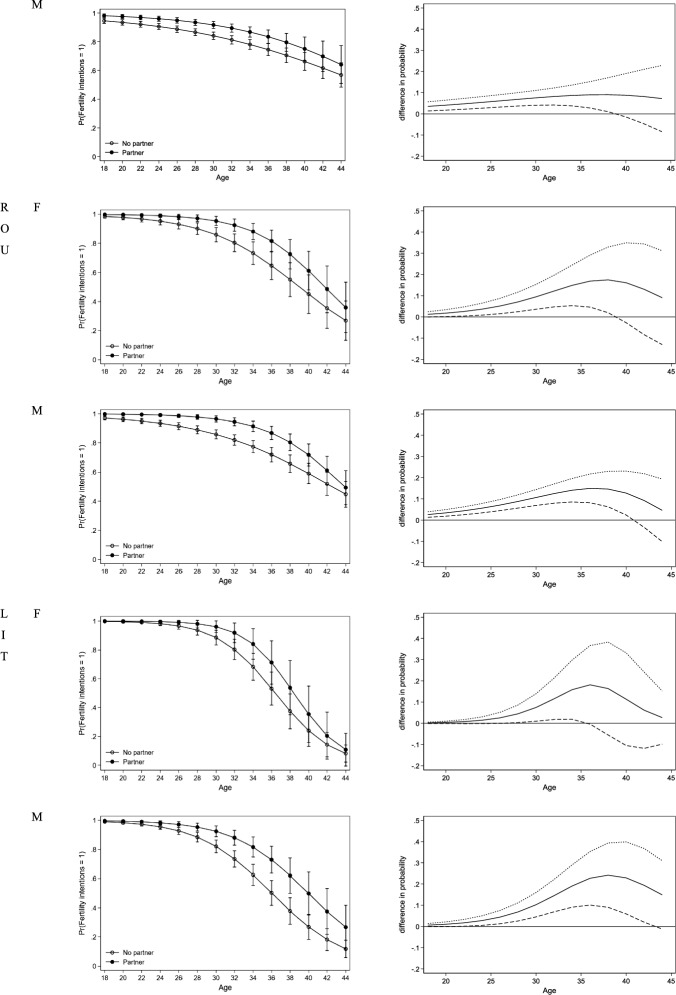

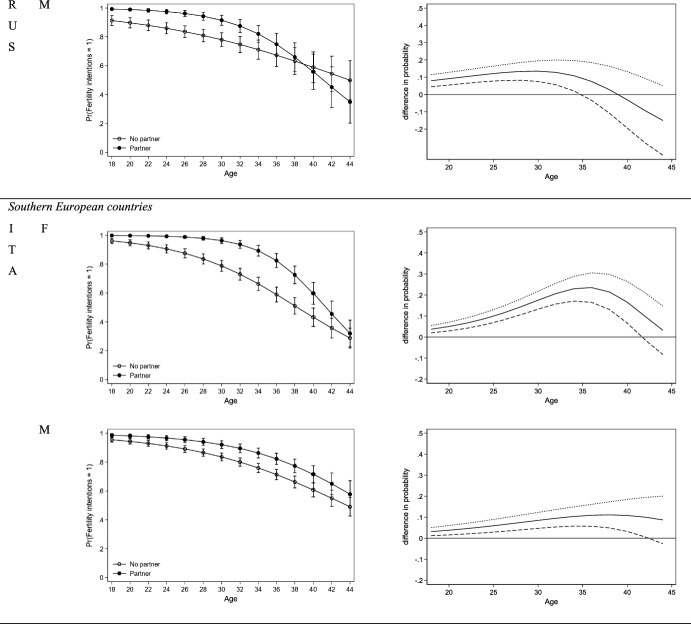
Predicted probability of intending to have (a) child(ren) (left) and difference in probability of fertility intention when being in a relationship (right) *Source*: Generations and gender services, wave 1; own calculations

M = Men, F = Women

## Data Availability

This article is based on data that are freely available to researchers on the conditions that the researchers in question are attached to a recognized research institute and the data are not used for commercial purposes.

## Appendix

### Overview of Missings on the Fertility Intention Variable Across Countries

The main variables of interest are relationship status and fertility intentions of men and women. To obtain reliable results, countries with high missings on the fertility variable were excluded from the analysis.

This is the case for men in Poland, where 61% of the respondents did not receive the question as well as in Norway, where 56% of the male respondents are missing. In Poland, about 90% of these respondents do not have a partner (*n* = 962) and in Norway, this is the case for 98% of the missings (*n* = 898). Since the difference between partnered and single respondents can therefore not be analysed, men are excluded from the analysis in these countries. Missings on fertility intentions among male respondents range from 25% in the Czech Republic, Belgium and the Netherlands to below 5% in the remaining countries. Again, the missings are biased in terms of the respondents who do not have a partner (about 90% of the missings) in the case of the Czech Republic (*n* = 336) and Belgium (*n* = 170) and therefore have to be excluded. The number of missings among men without a partner is also high in the Netherlands (*n* = 115), but in total, the number of missings is lower and 68% of these missings are among single men, which is comparably lower to the other countries. We decided to keep the male respondents of the Dutch sample in the analysis, but these issues have to be kept in mind when interpreting the results. Finally, 317 single men in Germany received the questions on fertility intentions but did not answer them. Since this makes up 63% of the single men in the sample, German male respondents are also excluded from the analysis.

Among the female respondents, high missings on fertility intentions can be found in Russia (63%), the Czech Republic (26%), the Netherlands (20%) and Poland (20%). Female respondents from the Russian, Czech Republican and Polish samples are excluded since about 80–90% of the missings are women without partners (except for Russia, where 70% of the missings are among the women in a relationship). The missings in the Dutch sample are comparably high in total but are evenly distributed among single and partnered women (50%). They are therefore kept in the sample. Finally, Estonia is not included in the analysis, since questions about fertility intentions were not asked to men and the sample size among women is low (for example, only 4 respondents have a partner and do not intend to have a child). Due to too low sample sizes, Georgia is also excluded from the sample.

See Tables 4, 5, 6, 7, 8, 9, 10

**Table 4:** Share in % of childless respondents with and without partner across age groups. *Source*: Generations and Gender survey, wave 1

Age	Coun.	Women		Men		Coun	Women		Men	
		No partner	Partner	No partner	Partner		No partner	Partner	No partner	Partner
Scandinavian countries										
< 25	NOR	50.4	49.6			SWE	50.4	49.6	60.4	39.6
25–29		38.0	62.0				33.0	67.0	40.9	59.1
30—34		43.0	57.1				28.7	71.3	33.8	66.2
> 34		46.5	53.5				32.3	67.7	55.2	44.8
Ø		45.9	54.1				42.3	57.7	52.0	48.0
Western European countries										
< 25	AUT	43.8	56.2	56.1	43.9	BEL	41.6	58.4		
25–29		29.6	70.4	32.9	67.1		32.9	67.2		
30–34		26.8	73.2	43.5	56.6		31.0	69.0		
> 34		38.1	62.0	40.8	59.2		42.5	57.5		
Ø		36.7	63.4	45.2	54.8		38.6	61.4		
< 25	FRA	43.1	57.0	59.2	40.8	DEU	34.8	65.2		
25—29		34.4	65.6	39.2	60.8		27.3	72.7		
30–34		48.3	51.7	55.4	44.6		25.5	74.5		
> 34		53.9	46.1	65.3	34.8		34.2	65.9		
Ø		43.9	56.1	55.8	44.2		31.5	68.5		
< 25	NLD	44.7	55.4	62.9	37.1					
25–29		31.0	69.0	40.3	59.7					
30–34		35.9	64.1	40.5	59.5					
> 34		45.6	54.4	48.0	52.0					
Ø		39.2	60.8	47.7	52.3					
Eastern European countries										
< 25	HUN	56.2	43.8	73.7	26.3	BGR	69.2	30.8	78.4	21.6
25–29		38.4	61.6	51.5	48.5		53.0	47.0	68.0	32.0
30–34		46.2	53.8	54.6	45.5		59.3	40.7	64.8	35.2
> 34		58.1	41.9	66.5	33.5		61.3	38.7	69.1	30.9
Ø		48.4	51.7	61.7	38.3		63.8	36.2	72.6	27.4
< 25	LTU	68.3	31.7	71.2	28.8	ROU	70.7	29.3	76.6	23.4
25–29		47.7	52.3	48.3	51.7		42.8	57.3	49.4	50.6
30—34		58.6	41.4	58.3	41.7		33.7	66.3	47.6	52.4
> 34		70.4	29.6	68.2	31.9		51.4	48.6	55.0	45.0
Ø		64.5	35.6	64.9	35.1		55.1	44.9	61.1	39.0
< 25	RUS			51.0	49.0					
25–29				42.2	57.8					
30–34				35.1	64.9					
> 34				49.6	50.4					
Ø				47.5	52.5					
Southern European country										
< 25	ITA	60.3	39.7	78.6	21.4					
25–29		43.9	56.1	59.6	40.4					
30–34		44.4	55.6	52.7	47.3					
> 34		55.3	44.7	64.9	35.1					
Ø		52.6	47.4	64.7	35.3

**Table 5:** Share in % of respondents intending to have children across countries, age groups, gender and partnership status for women and men. *Source*: Generations and Gender survey, wave 1

Age	Coun.	Women		Men		Coun	Women		Men	
		No partner	Partner	No partner	Partner		No partner	Partner	No partner	Partner
Scandinavian countries										
< 25	NOR	89.3	93.6			SWE	90.6	93.1	90.3	96.6
25—29		81.5	93.5				87.3	94.4	90.0	94.6
30—34		82.8	89.4				79.3	91.6	82.0	87.7
> 34		27.4	41.6				50.0	34.9	47.5	49.4
Ø		78.8	86.3				86.1	86.5	80.6	85.8
Western European countries										
< 25	AUT	88.4	92.1	86.4	92.5	BEL	82.0	84.2		
25–29		87.7	90.1	83.3	95.3		68.9	90.2		
30–34		81.5	83.6	74.6	91.4		63.6	75.5		
> 34		43.5	43.3	75.2	67.4		20.8	24.6		
Ø		79.0	81.4	82.1	87.4		65.4	74.3		
< 25	FRA	74.0	83.4	72.4	75.7	DEU	81.3	92.6		
25–29		79.5	94.2	71.8	88.4		90.0	85.1		
30–34		91.1	85.0	74.6	81.4		55.6	79.6		
> 34		32.0	39.8	42.9	42.7		17.0	15.1		
Ø		67.5	79.6	63.6	73.6		65.3	70.4		
< 25	NLD	89.5	95.8	91.0	94.9					
25–29		88.0	93.9	89.0	93.5					
30–34		78.5	74.0	75.0	85.1					
> 34		19.2	17.2	34.3	25.6					
Ø		68.7	74.6	69.9	70.6					
Eastern European countries										
< 25	HUN	93.4	96.3	90.1	92.5	BGR	93.4	99.1	90.8	92.4
25–29		92.1	98.0	89.9	95.4		91.5	99.2	92.2	99.1
30–34		88.1	92.3	81.8	91.8		89.0	90.0	86.4	96.2
> 34		40.0	55.6	57.2	73.8		54.7	76.7	65.7	81.8
Ø		87.1	93.8	83.3	91.1		88.1	95.1	86.7	93.0
< 25	LTU	98.8	99.3	97.7	98.4	ROU	96.2	98.7	93.3	98.1
25–29		96.8	100.0	92.2	100.0		92.8	98.7	95.9	96.9
30–34		91.2	95.8	76.2	91.1		87.8	95.4	86.1	97.7
> 34		26.3	31.2	20.6	42.0		46.4	50.9	49.4	67.9
Ø		87.3	91.5	84.2	91.2		86.5	88.6	82.7	88.9
< 25	RUS			86.4	98.1					
25—29				90.0	96.9					
30—34				84.6	85.4					
> 34				50.0	55.7					
Ø				81.1	90.1					
Southern European country										
< 25	ITA	88.3	98.0	89.2	91.6					
25–29		93.9	97.9	89.7	94.9					
30–34		84.7	95.6	88.1	93.7					
> 34		37.2	53.9	53.7	65.7					
Ø		71.8	84.4	77.6	84.7

**Table 6:** Average marginal effect of having a partner on fertility intentions (with age as squared and continuous variable). *Source*: Generations and Gender survey, wave 1; own calculations

Coun.	M/F	Partner	95%-Confidence interval	
Scandinavian countries				
NOR	F	0.075***	0.0382	0.111
SWE	F	0.031	− 0.010	0.072
	M	0.042*	0.003	0.081
Western European countries				
AUT	F	0.021	− 0.022	0.064
	M	0.045*	0.001	0.089
BEL	F	0.068*	0.038	0.131
DEU	F	0.067*	0.011	0.124
FRA	F	0.072**	0.024	0.120
	M	0.053 +	− 0.004	0.110
NLD	F	0.022	− 0.025	0.070
	M	− 0.010	− 0.060	0.040
Eastern European countries				
BUL	F	0.068***	0.041	0.095
	M	0.059***	0.029	0.089
HUN	F	0.044**	0.013	0.075
	M	0.061***	0.028	0.093
ROU	F	0.057**	0.016	0.099
	M	0.061**	0.026	0.095
LIT	F	0.029*	0.002	0.056
	M	0.053***	0.025	0.081
RUS	M	0.077***	0.033	0.121
Southern European countries				
ITA	F	0.113***	0.078	0.148
	M	0.062**	0.026	0.098

Average marginal effect; M = Men; F = Women

Significance stars

**Table 7:** Results logistic regression: Effect of partnership, age (categorized) and educational level on intention to have children. *Source*: Generations and Gender survey, wave 1; own calculations

Coun.	M/F	Partner(ref.: no partner)	Age				Educational level
			< 25	25–29	Ref: 30–34	> 35	
Scandinavian countries							
NOR	F	2.050***	2.092*	1.233	1.000	0.084***	1.007
SWE	F	1.308	2.217*	1.675	1.000	0.090***	1.019 +
	M	1.458*	3.392***	3.392***	1.000	0.178***	1.027**
Western European countries							
AUT	F	1.180	2.414**	1.791*	1.000	0.166***	1.017**
	M	1.486*	1.972*	1.985*	1.000	0.452**	1.019**
BEL	F	1.610*	2.448**	1.900 +	1.000	0.126***	1.016*
DEU	F	1.639*	3.474***	2.882**	1.000	0.076***	1.019*
FRA	F	1.682**	0.521*	1.045	1.000	0.077***	1.003
	M	1.338 +	0.831	1.208	1.000	0.229***	1.006
NLD	F	1.184	4.596***	3.795***	1.000	0.074***	1.004
	M	0.989	3.520***	2.741**	1.000	0.107***	1.019***
Eastern European countries							
BUL	F	3.065***	3.774***	2.175 +	1.000	0.200***	1.028***
	M	2.140***	1.635 +	2.097*	1.000	0.279***	1.034***
HUN	F	2.160**	2.523*	2.067 +	1.000	0.088***	1.029***
	M	1.878***	1.828*	1.841*	1.000	0.289***	1.026***
ROU	F	1.871*	3.594*	1.840	1.000	0.076***	1.018*
	M	2.248***	1.950 +	2.544*	1.000	0.123***	1.014 +
LIT	F	1.616	9.400**	3.942	1.000	0.027***	1.016
	M	2.737***	15.244***	5.021***	1.000	0.085***	1.026**
RUS	M	2.338***	2.371*	2.934*	1.000	0.209***	1.013
Southern European countries							
ITA	F	2.505***	1.490	2.592*	1.000	0.083***	1.016***
	M	1.704***	1.092	1.169	1.000	0.157***	1.020***

Odds ratios; M = Men; F = Women

Significance stars

**Table 8:** Results logistic regression: Effect of partnership (ref.: no partner), age (continuous), age (squared) and educational level on intention to have children *Source*: Generations and Gender survey, wave 1; own calculations

Coun.	M/F	Partner	Age	Age (squared)	Education
Scandinavian countries					
NOR	F	2.134***	1.101 +	0.989***	1.005
SWE	F	1.415	1.073	0.990***	1.014
	M	1.495*	0.888**	0.999	1.026**
Western European countries					
AUT	F	1.195	1.039	0.993***	1.015*
	M	1.467*	1.059	0.994**	1.017**
BEL	F	1.585*	1.094	.0.990***	1.012 +
DEU	F	1.710*	0.996	0.992***	1.021**
FRA	F	1.632**	1.269***	0.987***	1.000
	M	1.340 +	1.164***	0.992***	1.005
NLD	F	1.240	1.112	0.985***	1.004
	M	0.914	0.969	0.993**	1.021***
Eastern European countries					
BUL	F	3.291***	0.920 +	0.997 +	1.033***
	M	2.137**	1.043	0.995***	1.035***
HUN	F	2.007**	1.037	0.991**	1.030***
	M	1.879**	0.995	0.997*	1.024***
ROU	F	2.461**	0.938	0.995*	1.016 +
	M	2.150**	1.028	0.994***	1.019*
LIT	F	2.349*	0.830 +	0.995	1.025*
	M	2.654**	0.775***	1.000	1.028**
RUS	M	2.278**	1.029	0.994**	1.014 +
Southern European countries					
ITA	F	3.227***	1.098*	0.990***	1.018**
	M	1.712**	1.067*	0.994***	1.023***

Odds ratios; M = Men; F = Women

Significance stars

**Table 9:** Average marginal effect of having a partner on fertility intentions (with age as continuous variable) *Source*: Generations and Gender survey, wave 1; own calculations

Coun.	M/F	Partner	95%-Confidence interval	
Scandinavian countries				
NOR	F	0.082***	0.044	0.119
SWE	F	0.039+	− 0.004	0.081
	M	0.044*	0.005	0.082
Western European countries				
AUT	F	0.029	− 0.014	0.074
	M	0.051*	0.007	0.095
BEL	F	0.084*	0.019	0.159
DEU	F	0.075*	0.017	0.132
FRA	F	0.090***	0.040	0.141
	M	0.075*	0.017	0.134
NLD	F	0.030	− 0.019	0.080
	M	− 0.004	− 0.055	0.047
Eastern European countries				
BUL	F	0.070***	0.043	0.097
	M	0.063***	0.034	0.093
HUN	F	0.051**	0.019	0.083
	M	0.066***	0.034	0.098
ROU	F	0.064**	0.024	0.106
	M	0.073***	0.038	0.109
LIT	F	0.029*	0.001	0.057
	M	0.053***	0.025	0.081
RUS	M	0.088***	0.043	0.133
Southern European countries				
ITA	F	0.119***	0.082	0.156
	M	0.076***	0.040	0.112

Average marginal effect; M = Men; F = Women

Significance stars

## Footnotes

1 See Appendix for an overview of the missing values.

2 In Hungary, Norway and the Netherlands the questions can be answered only with “Yes” or “No”.

3 Measuring age as a squared variable (Table 3) or as a continuous variable (Table 6 in the Appendix) yields similar results aside from small differences in the coefficients.

4 Additionally, we ran our analysis measuring partnership status with three instead of two categories: Partnered, Single (cohabited before), Single (never cohabited). In France, Belgium and Italy, singles that never cohabited are less likely to desire a child than singles who previously cohabited. However, this relationship is statistically significant only in a very short age window for all three countries. In the majority of countries there are no significant statistical differences (possibly also due to the reduced sample size).

## Code Availability

The code is stored at the Netherlands Interdisciplinary Demographic Institute. In case of doubt about the replicability of the results of this article, the Netherlands Interdisciplinary Demographic Institute can be contacted.
